# Proportion of neonatal readmission attributed to length of stay for childbirth: a population-based cohort study

**DOI:** 10.1136/bmjopen-2016-012007

**Published:** 2016-09-14

**Authors:** Amy Metcalfe, Matthews Mathai, Shiliang Liu, Juan Andres Leon, K S Joseph

**Affiliations:** 1 Department of Obstetrics and Gynaecology, University of Calgary, Calgary, Alberta, Canada; 2 Department of Maternal, Newborn, Child and Adolescent Health, World Health Organization, Geneva, Switzerland; 3 Liverpool School of Tropical Medicine, Liverpool, UK; 4 Maternal, Child and Youth Health, Surveillance and Epidemiology Division, Centre for Chronic Disease Prevention, Public Health Agency of Canada, Ottawa, Ontario, Canada; 5 Department of Obstetrics and Gynecology, University of British Columbia, Vancouver, British Columbia, Canada

**Keywords:** NEONATOLOGY, EPIDEMIOLOGY, PAEDIATRICS

## Abstract

**Objective:**

Most literature on length of stay (LOS) for childbirth focuses on ‘early’ discharge as opposed to ‘optimal’ time of discharge and has conflicting results due to heterogeneous definitions of ‘early’ discharge and differing eligibility criteria for these programmes. We aimed to determine the LOS associated with the lowest neonatal readmission rate following childbirth by examining the incidence pattern of neonatal readmission for different LOS using the Kitagawa decomposition.

**Design:**

Retrospective cohort study using administrative hospitalisation data.

**Setting:**

Canada (excluding Quebec) from 2003 to 2010.

**Patients:**

Term, singleton live births without congenital anomalies.

**Interventions:**

LOS for childbirth.

**Main outcome measure:**

Neonatal readmissions within 30 days of birth.

**Results:**

1 875 322 live births were included. Neonatal LOS peaked at day 1 (47.3%) after vaginal birth and day 3 (49.3%) following caesarean section; 4.2% of infants were readmitted following vaginal birth and 2.2% after caesarean section. In 2008–2010, most readmissions occurred among infants discharged in the first 2 days (83.8%) following a vaginal birth and among infants discharged in the first 3 days (81.7%) following a caesarean birth. Readmissions increased from 4.1% in 2003–2005 to 4.6% in 2008–2010 among vaginal births and from 2.0% to 2.4% among caesarean births and occurred mostly due to changes in the day-specific readmission rates and not due to reductions in LOS.

**Conclusions:**

Patterns of readmission suggest that readmission rates are lowest following a 1–2-day stay following a vaginal birth and a 2–4-day stay following a caesarean birth given the outpatient support in the community.

**Strengths and limitations of this study**

- This study was national in scope; it evaluated all lengths of stay in all hospitals (not just early discharge programmes targeted at a subset of the population) and readmissions to any hospital, not just the delivery hospital.
- The data for this study come from a population-based data set that has been validated to study perinatal events.
- This study only evaluates the impact of length of stay on neonatal admission rates. Multiple other outcomes are important in assessing the length of stay for childbirth associated with the fewest neonatal readmissions.
- Length of stay was calculated in days not hours, limiting our ability to examine very short lengths of stay (ie, <12 hours) in finer detail.
- Limited or no data were available on both individual-level and area-level confounders such as regional variability in community-based supports available, breast feeding and use of additional outpatient health services, which may have influenced readmission rates.

## Introduction

Hospitalisation for childbirth is one of the most frequent categories of hospital admission in industrialised countries.^1^ ^2^ Numerous studies have reported that the length of stay (LOS) for childbirth has been steadily decreasing in recent decades, in an effort to decrease costs and demedicalise pregnancy.^1^ ^3–5^ The medical necessity of hospitalisation for and after childbirth is influenced by a variety of factors such as the availability of follow-up services, the organisation of maternity care, the medical and social needs of mother and infant, mode of delivery and parity.^2^ ^5–9^ In Canada, over 98% of births occur in a hospital setting;^10^ ^11^ mothers and infants are typically hospitalised together prior to discharge. Approximately 12.1% of infants and 0.2% of mothers require treatment in an intensive care unit during this initial hospitalisation.^12^ Transfer of the mother or infant (or both) to an intensive care unit may result in the separation of mother and infant and typically results in a longer LOS.^12^ Following discharge, infants are seen routinely in the community by physicians and public health nurses for a series of well-baby visits and vaccinations during the first year of life.

Most of the literature on LOS for childbirth has evaluated the impact of ‘early discharge’ with conflicting results. Some studies have shown that early discharge does not impact infant readmission,^13–16^ while others have demonstrated an increase in infant readmission after early discharge.^17^ ^18^ However, synthesis of this body of literature is complicated by differing definitions of ‘early’ discharge (<24, <36, <48, <72 hours), differing time periods for readmission (7–90 days), the availability of non-hospital-based support systems, and the generalisability to all pregnant women, not just those meeting strictly defined criteria.^14^ ^15^ ^19^ ^20^ Additionally, while understanding the impact of early discharge on maternal and infant health is a valid concern, the focus needs to shift from evaluating the impact of ‘early discharge’ to determining the LOS associated with the least readmissions following childbirth.^3^

Readmissions are an important outcome for evaluating the optimal LOS following childbirth as they are typically measures of severe morbidity. While some readmissions may be precautionary to monitor the infant for suspected disease and some are preventable, reducing readmissions is an important element of high-quality care.^21^ Furthermore, readmissions are costly and may be a direct consequence of a reduced LOS (due to insufficient time to observe the patient for latent signs of disease or insufficient instruction on proper newborn care^22^). It is estimated that ∼3% of infants will be readmitted to hospital.^23^ The most frequently reported causes of infant readmission are: dehydration, diarrhoea, feeding problems, fever, infections, gastrointestinal problems, jaundice, sepsis and viral/respiratory issues.^4^ ^13^ ^15^ ^16^ ^20^ ^24^ This study aimed to determine what proportion of neonatal readmissions can be attributed to changing LOS for childbirth and to identify the LOS for childbirth associated with the lowest readmission rate.

## Methods

The study population included all singleton live births in Canada (excluding Quebec) from 2003 to 2010 with data on LOS and readmissions obtained from the Discharge Abstract Database (DAD) of the Canadian Institute for Health Information. Live births were identified by the use of International Classification of Diseases (ICD-10-CA) code Z37.0 (singleton live birth). This study was limited to all hospital deliveries of live-born infants between 2003 and 2010 where the mother and infant are both discharged from hospital on the same day to minimise the impact of severe maternal or neonatal complications requiring an extended LOS. In the presence of extended LOS for neonatal indications, it would be very rare for the mother to remain in hospital. This study period was chosen as all records in the database during this period were coded with ICD-10-CA. Mother–infant dyads that included infants with congenital anomalies (ICD-10-CA Q00-Q99) identified at birth, multiple gestation pregnancies (O30.0, O30.1, O30.2, O30.8, O30.9, O84.0, O84.9), premature births (gestational age <37 weeks) and maternal deaths were excluded.

LOS was derived by subtracting the date of birth from the date of discharge and was used as a continuous variable. Transfers between hospitals or between units within a single hospital were counted as a single admission. Mode of delivery was classified as vaginal or caesarean (Canadian classification of health interventions code 5MD60). Neonatal readmissions were examined within the first 30 days of life. Neonatal readmissions could occur in either the same hospital where the delivery occurred or another hospital in Canada and could also occur in regular or special care units. All-cause and cause-specific readmission for confirmed jaundice (ICD-10-CA P55-P59), infection (ICD-10-CA P35-P39) and dehydration (ICD-10-CA P74.1) were evaluated.

The Kitagawa decomposition was used to assess the impact of temporal changes in the LOS and temporal changes in the LOS-specific readmission rate on overall temporal changes in all-cause and cause-specific readmission rates. Separate equations were used for vaginal and caesarean births. The Kitagawa decomposition formula is given below:1
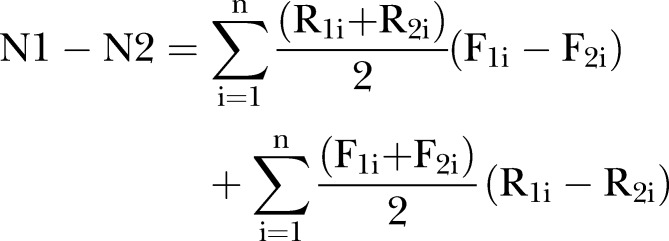


N refers to the neonatal readmission rate in periods 1 (2003–2005) and 2 (2008–2010); R represents the neonatal readmission rate for a given LOS (i), while F indicates the proportion of infants with a given LOS (i). The first part of this equation quantifies the impact of temporal changes in LOS on temporal changes in neonatal readmission rates and aims to answer the question: ‘Are neonatal readmission rates increasing because LOS is decreasing?’ while the second part of the equation quantifies the impact of temporal changes in LOS-specific (or day-specific) neonatal rates on temporal changes in neonatal readmission rates and aims to answer the question: ‘Are neonatal readmission rates increasing because the threshold for admission is getting lower?’ All analyses were conducted using SAS V.9.2.

## Results

There were 490 125 singleton live births delivered vaginally in 2003–2005, of which 19 547 were readmitted, yielding a readmission rate of 3.99 per 100 live births. Among 160 038 singleton live births delivered by caesarean in 2003–2005, there were 3460 readmissions (readmission rate 2.16 per 100 live births). In 2008–2010, there were 611 342 live births and 27 567 readmissions (readmission rate 4.51 per 100 live births) following vaginal birth and 221 642 live births and 5700 readmissions (readmission rate 2.57 per 100 live births) following a caesarean birth. In 2008–2010, most readmissions occurred among infants discharged in the first 2 days (83.8%) or the first 3 days (94.5%) following a vaginal birth and among infants discharged in the first 3 days (81.7%) or the first 4 days (93.3%) after a caesarean birth.

**Figure 1:**
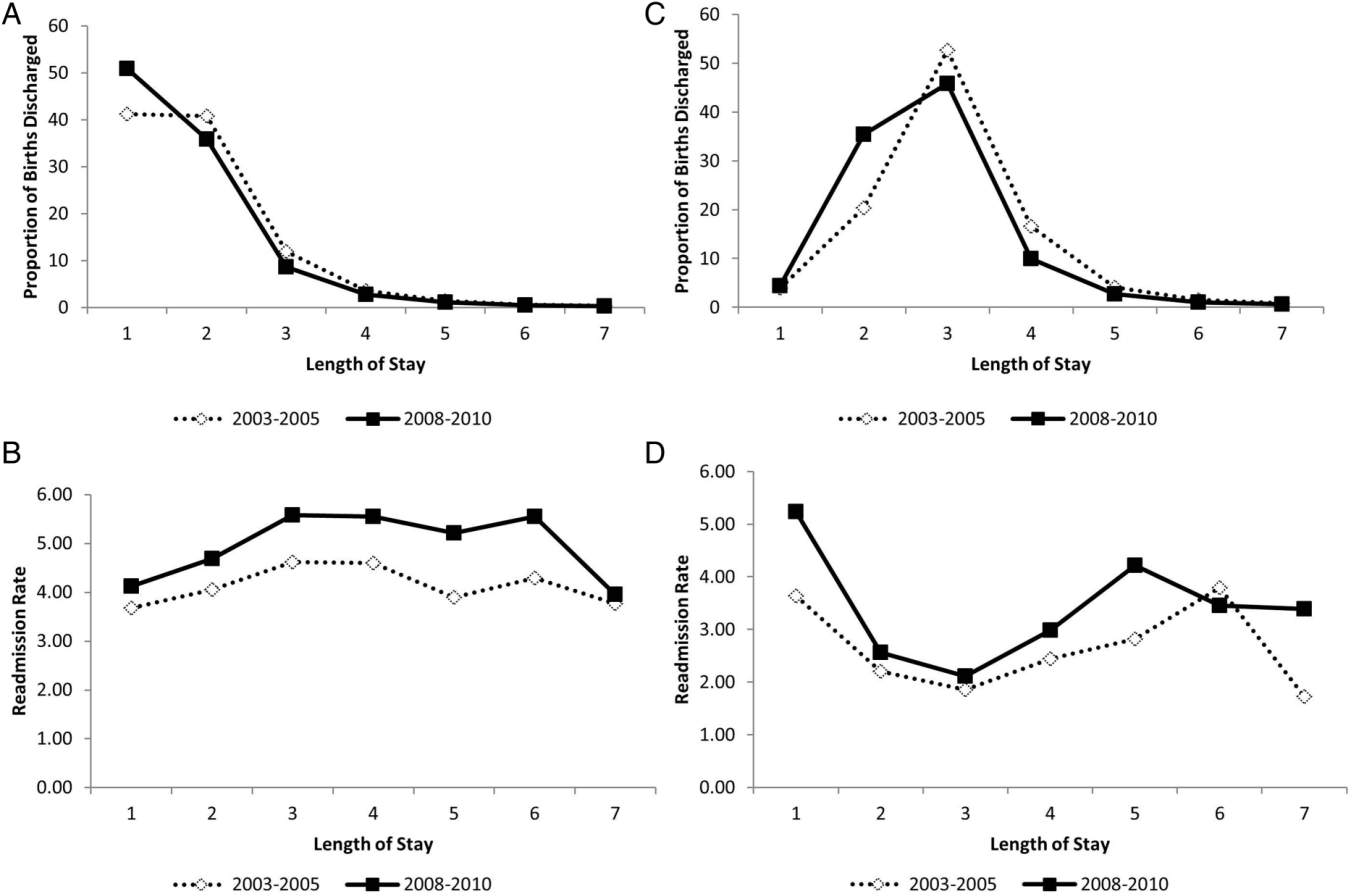
Temporal changes in length of stay (A and C) and readmission rates (B and D) between 2003 and 2005 and 2008 and 2010 for vaginal (A and B) and caesarean births (C and D) for singleton live births in Canada (excluding Quebec).

LOS for childbirth decreased while neonatal readmission rates increased between 2003 and 2005 and 2008 and 2010 for vaginal and caesarean births (figure 1). In 2003–2005, the largest proportion of infants were discharged on day 1 (41.2%, 95% CI 41.1% to 41.4%), day 2 (40.8%, 95% CI 40.7% to 41.0%) or day 3 (11.9%, 95% CI 11.8% to 12.0%) following a vaginal birth. By 2008–2010, the timing of discharge had shifted with an increasing proportion of infants being discharged on day 1 (50.9%, 95% CI 50.8% to 51.1%), day 2 (35.8%, 95% CI 35.7% to 35.9%) and day 3 (8.7%, 95% CI 8.6% to 8.7%) following a vaginal birth. Overall readmission rates following a vaginal birth were 13% higher in 2008–2010 compared with 2003–2005 (table 1).

**Table 1:** Neonatal readmission by length of stay 2003–2005 vs 2008–2010

Mode of delivery	Length of stay (days)	2003–2005		2008–2010		Rate difference	Rate ratio (95% CI)
		N (%)	Neonatal readmission rate per 100 live singleton births	N (%)	Neonatal readmission rate per 100 live singleton births		
Vaginal	1	7442 (38.1)	3.68	12 831 (46.5)	4.12	0.44	1.12 (1.09 to 1.15)
	2	8119 (41.5)	4.06	10 272 (37.3)	4.69	0.63	1.16 (1.12 to 1.19)
	3	2701 (13.8)	4.62	2960 (10.7)	5.58	0.97	1.21 (1.15 to 1.27)
	4	813 (4.2)	4.61	921 (3.3)	5.55	0.95	1.21 (1.10 to 1.32)
	5	268 (1.4)	3.90	339 (1.2)	5.21	1.32	1.34 (1.14 to 1.57)
	6	130 (0.7)	4.30	168 (0.6)	5.55	1.25	1.29 (1.03 to 1.62)
	7	74 (0.4)	3.76	76 (0.3)	3.96	0.20	1.05 (0.76 to 1.45)
	Overall	19 547 (100.0)	3.99	27 567 (100.0)	4.51	0.52	1.13 (1.11 to 1.15)
Caesarean	1	227 (6.6)	3.64	510 (8.9)	5.23	1.59	1.44 (1.23 to 1.68)
	2	720 (20.8)	2.21	2006 (35.2)	2.56	0.35	1.16 (1.07 to 1.26)
	3	1566 (45.3)	1.86	2143 (37.6)	2.11	0.25	1.14 (1.06 to 1.21)
	4	648 (18.7)	2.44	659 (11.6)	2.98	0.54	1.22 (1.10 to 1.36)
	5	184 (5.3)	2.82	252 (4.4)	4.21	1.40	1.50 (1.24 to 1.81)
	6	92 (2.7)	3.79	80 (1.4)	3.45	−0.34	0.91 (0.67 to 1.23)
	7	23 (0.7)	1.72	50 (0.9)	3.39	1.67	1.97 (1.20 to 3.22)
	Overall	3460 (100.0)	2.16	5700 (100.0)	2.57	0.41	1.19 (1.14 to 1.24)

A similar pattern emerged for caesarean births. In 2003–2005, the majority of infants were discharged on day 2 (20.4%, 95% CI 20.2% to 20.6%), day 3 (52.7%, 95% CI 52.4% to 52.9%) or day 4 (16.6%, 95% CI 16.4% to 16.8%) following a caesarean birth. By 2008–2010, the timing of discharge had shifted with an increasing proportion of infants being discharged on day 2 (35.4%, 95% CI 35.2% to 35.6%), day 3 (45.8%, 95% CI 45.6% to 46.0%) or day 4 (10.0%, 95% CI 9.9% to 10.1%) following a caesarean birth. Overall readmission rates following a caesarean birth were 19% higher in 2008–2010 compared with 2003–2005 (table 1).

The majority of neonatal readmissions were for jaundice (49.9%), respiratory conditions (8.1%), feeding problems (5.2%), sepsis (4.0%) and dehydration (3.3%). Overall readmission rates for specific conditions were significantly higher following vaginal births compared with caesarean births (ie, the readmission rate for jaundice was 2.11% (95% CI 2.09% to 2.13%) following vaginal births compared with 0.91% (95% CI 0.88% to 0.93) following caesarean births. No temporal trends were observed among readmissions for infections and dehydration; however, readmission rates for jaundice increased over time.

The Kitagawa decomposition for readmission rates following vaginal birth showed that most of the rate difference in readmission rates between 2003 and 2005 and 2008 and 2010 (0.52 readmissions per 100 live births) was due to changes in the day-specific readmission rates and not due to changes in LOS (table 2). The greatest change was observed following an LOS of 1 day. For an LOS of 1 day following a vaginal birth, the rate difference is jointly attributed to changing LOS (65.10%) and day-specific readmission rates (34.90%). However, overall, these changes in LOS were overwhelmed by changes in the day-specific readmission rate. Of the total rate difference of 51.87 per 10 000 live births, −7.78 per 10 000 live births were due to changes in LOS and 59.65 per 10 000 live births were attributed to changes in day-specific readmission rates. Similarly, the Kitagawa decomposition attributed the rate difference in readmission rates following caesarean birth between 2003 and 2005 and 2008 and 2010 (0.41 per 100 live births) to changes in day-specific readmission rates (and not due to changes in LOS).

**Table 2:** Changes in overall neonatal readmission rates (2003–2005 vs 2008–2010) attributable to temporal changes in length of stay and length of stay specific readmission rates

Mode of delivery	Length of stay (days)	Contribution of changes in		Total change	Relative contribution of changes in	
		Length of stay	Length of stay specific neonatal readmission		Length of stay (%)	Length of stay specific neonatal readmission (%)
Vaginal	1	37.83	20.28	58.11	65.10	34.90
	2	−21.88	24.14	2.26	−968.14	1068.14
	3	−16.68	9.89	−6.79	245.66	−145.66
	4	−4.52	2.97	−1.55	291.61	−191.61
	5	−1.55	1.61	0.06	−2583.33	2683.33
	6	−0.64	0.69	0.05	−1280.00	1380.00
	7	−0.35	0.07	−0.28	125.00	−25.00
	Total	−7.78	59.65	51.87	−15.00	115.00
Caesarean	1	2.22	6.60	8.82	25.17	74.83
	2	35.73	9.76	45.49	78.54	21.46
	3	−13.60	12.31	−1.29	1054.26	−954.26
	4	−18.46	7.12	−11.34	162.79	−62.79
	5	−4.85	4.71	−0.14	3464.29	−3364.29
	6	−1.70	−0.44	−2.14	79.44	20.56
	7	−0.41	1.25	0.84	−48.81	148.81
	Total	−1.07	41.32	40.25	−2.65	102.65

The above table highlights differences in the overall neonatal readmission rate between 2003 and 2005 and 2008 and 2010 (among singleton live births in Canada, excluding Quebec) that are attributed to either changes in neonatal length of stay or readmission rates for a given length of stay. For example, the excess 2.26 neonatal readmissions for a length of stay of 2 days following a vaginal birth occurred entirely due to temporal differences in the length of stay specific readmission rate and not due to temporal changes in length of stay.

Table 3 shows the results of the Kitagawa decomposition for readmissions due to jaundice. The rate difference in readmission rates for jaundice following vaginal birth between 2003 and 2005 and 2008 and 2010 was entirely due to changes in day-specific readmission rates (and not due to changes in LOS), while the rate difference in readmission rates following caesarean birth was mostly due to changes in day-specific readmission rates (86.1%) and partly due to changes in the LOS (13.9%).

**Table 3:** Changes in neonatal readmission rates for jaundice (2003−2005 vs 2008−2010) attributable to temporal changes in length of stay and length of stay specific readmission rates

Mode of delivery	Length of stay (days)	Contribution of changes in		Total change	Relative contribution of changes in	
		Length of stay	Length of stay specific neonatal readmission		Length of stay (%)	Length of stay specific neonatal readmission (%)
Vaginal	1	20.37	16.59	36.96	55.11	44.89
	2	−10.95	18.39	7.44	−147.18	247.18
	3	−6.70	5.36	−1.34	500.00	−400.00
	4	−1.94	2.46	0.52	−373.08	473.08
	5	−0.68	0.81	0.13	−523.08	623.08
	6	−0.23	0.20	−0.03	766.67	−666.67
	7	−0.10	0.12	0.02	−500.00	600.00
	Total	−0.23	43.93	43.70	−0.53	100.53
Caesarean	1	1.01	1.95	2.96	34.12	65.88
	2	15.35	3.63	18.98	80.87	19.13
	3	−5.00	6.90	1.90	−263.16	363.16
	4	−6.03	4.35	−1.68	358.93	−258.93
	5	−1.54	2.61	1.07	−143.93	243.93
	6	−0.46	0.93	0.47	−97.87	197.87
	7	−0.08	−0.20	−0.28	28.57	71.43
	Total	3.25	20.17	23.42	13.88	86.12

The above table highlights differences in the neonatal readmission rate for jaundice between 2003 and 2005 and 2008 and 2010 (among singleton live births in Canada, excluding Quebec) that are attributed to either changes in neonatal length of stay or readmission rates for a given length of stay. For example, the excess 7.44 neonatal readmissions for a length of stay of 2 days following a vaginal birth occurred entirely due to temporal differences in the length of stay specific readmission rate and not due to temporal changes in length of stay.

## Discussion

Our study shows that LOS following childbirth decreased while neonatal readmission rates increased between 2003 and 2005 and 2008 and 2010 for vaginal and caesarean births. Overall and indication-specific readmission rates were significantly higher following vaginal births; this is consistent with what has been observed in other settings.^25^ ^26^ The reasons for this are most likely twofold; first, a higher proportion of infants are discharged on day 1 following a vaginal birth compared with a caesarean birth (and following an LOS of 1 day, readmission rates are similar between infants born vaginally or via caesarean section). Second, since it is common practice in Canada for infants born following a vaginal birth to be discharged on day 1 or 2 and for infants born following a caesarean section to be discharged on day 3 or 4, infants who were born vaginally and still hospitalised on day 3 were most likely not as healthy as infants born following a caesarean section who had a similar LOS. However, neonatal readmission rates remain low overall, even though neonatal readmission rates have increased over time in Canada. The increase in readmission rates during this period was almost entirely due to changes in day-specific readmission rates and not due to changes in LOS. This was true for overall rates of readmission following childbirth and also for readmission for jaundice. In 2008–2010, most readmissions occurred among infants discharged in the first 2 days (83.8%) following a vaginal birth and among infants discharged in the first 3 days (81.7%) after a caesarean birth. A slightly higher relative increase in readmission rates was observed following caesarean births compared with vaginal births. The reasons for this are unknown, but may be due to increased obstetric intervention in the early term period (ie, 37–38 weeks of gestation).^27^ The rate of caesarean sections steadily increased over the study period from 25.8% in 2003/2004 to 27.8% in 2009/2010,^28^ and elective early term repeat caesarean sections are common—a study from one Canadian province found that between 2008 and 2011, 55% of elective repeat caesarean sections occurred prior to 39 weeks of gestation.^29^

There are advantages and disadvantages associated with a shorter LOS following childbirth. Advantages of a shorter LOS include decreased costs, improved attachment and improved breastfeeding rates in a family setting,^13^ ^17^ while disadvantages include less time to observe the mother and the infant for latent medical problems, less time for education on infant care, and less time to initiate and establish breast feeding.^13^ Our study suggests that neonatal readmission rates during recent years are not increasing due to decreasing LOS, but instead due to changes in the day-specific readmission rates, that is, a lowering of the threshold for readmission. This is further supported by the fact that the highest relative increases in readmission rates were observed in infants with a long initial LOS (5 or 6 days following a vaginal birth and 5 or 7 days following a caesarean birth). Based on the observed readmission patterns, the time of discharge associated with the fewest neonatal readmissions appears to be after 1–2 days of hospital stay following a vaginal birth and 2–4 days following a caesarean birth, as this would avert the vast majority of readmissions. An Australian study examining neonatal readmissions for jaundice found similar results—the majority of readmissions occurred between days 3 and 6; however, at 37 and 38 weeks of gestation, respectively, 31 and 83 infants would need to have an initial LOS of 3 or more days to prevent a single admission for jaundice.^27^ The advantages of a shorter LOS may be realised given appropriate community support. While the Society of Obstetricians and Gynecologists of Canada (SOGC) has issued explicit criteria for postpartum discharge <48 hours after birth,^23^ they have not articulated what they deem to be the optimal time for discharge following childbirth. Regardless of the LOS following childbirth, the third and fourth days postbirth have been deemed to be a critical period during which all mothers and infants should be evaluated by a health professional.^3^

A nine-country European study showed a substantial intercountry variation in maternal average LOS following normal delivery (defined as vaginal birth of a singleton infant at term with no complications) ranging from 0.86 days in the Netherlands to 4.9 days in France.^6^ Variations in LOS contribute to the wide fluctuations in the cost of childbirth between and within countries.^6^ A policy analysis of hospital costs in the UK concluded that simply reducing the LOS may not result in appreciable cost-savings unless staffing levels are also reduced (and cautioned that a reduction in staffing levels may result in decreased quality of care).^30^ Many studies support the proposition that the timing of discharge can be individualised based on the health of the mother and infant and the resources available to them in their local community. An American study found that infants who had an outpatient well-baby visit in their community shortly after hospital discharge were significantly less likely to be readmitted for jaundice.^31^ An Italian study of an individualised early discharge and follow-up programme of term neonates resulted in no readmissions for jaundice or dehydration in the first 28 days of life.^32^ A Canadian study determined that in spite of only 4% of infants requiring readmission postbirth, this was the single greatest cost to the healthcare system in the first month after birth.^33^

This study has several strengths and some limitations. Since readmission postbirth is a relatively uncommon outcome, we had a sufficiently large sample size to achieve adequate statistical power.^15^ ^20^ ^22^ Additionally, we examined data from the population of healthy term singleton infants (excluding those with congenital anomalies), not a restricted subset of the population that might be eligible for early discharge programmes. Despite restricting our sample to apparently healthy singleton term infants, we cannot rule out the possibility of confounding by indication as the reason some infants have longer LOS might be the same reason why they are readmitted. This might result in an alternative explanation of the second part of the Kitagawa decomposition whereby instead of the threshold for readmission getting lower, infants’ health status may be lower in 2008–2010 (perhaps because of maternal morbidities), or a combination of these factors. We also identified our population through the use of a single ICD-10-CA code for singleton live births. A US study found that the use of a single ICD code to identify deliveries missed 3.4% of births, particularly those associated with severe obstetric complications.^34^ Since the objective of this study was to identify healthy newborns that were discharged on the same day as their mother, the impact of different case definitions to identify births is believed to be minimal. Additionally, we had no data on community resources and use of outpatient services after discharge. This is important as in some jurisdictions LOS for childbirth decreased in the late 1980s/early 1990s, concomitantly with reductions in postpartum home visits by public health nurses.^35^ Such simultaneous decreases in LOS following childbirth and reductions in community support following hospital discharge contribute to increasing readmission rates of infants.^35^ We were unable to differentiate between preventable readmissions and essential readmissions. A Canadian study from Alberta found that potentially preventable readmissions related to jaundice, dehydration, feeding problems, weight gain or social reasons occurred following 3.5% of deliveries and that over 80% of these occurred within the first week following discharge.^36^

Patterns of readmission suggest that readmission rates are lowest following a 1–2-day stay following a vaginal birth and a 2–4-day stay following a caesarean birth given that community-level supports are available, such as the case in Canada. However, contextual factors need to be considered when determining the optimal LOS for a particular infant. The low readmission rates observed in Canada and the varying LOS for apparently healthy neonates indicate that healthcare providers do a good job of risk stratifying infants. Integration of inpatient and outpatient services is critical to ensure that neonates receive the appropriate follow-up care in the community. Future studies should examine geographic variability in community support for childbirth and childrearing and its relationship with readmission rates and child health.
